# Both chimpanzee adenovirus-vectored and DNA vaccines induced long-term immunity against Nipah virus infection

**DOI:** 10.1038/s41541-023-00762-3

**Published:** 2023-11-04

**Authors:** Mingqing Lu, Yanfeng Yao, Xuekai Zhang, Hang Liu, Ge Gao, Yun Peng, Miaoyu Chen, Jiaxuan Zhao, XiaoYu Zhang, Chunhong Yin, Weiwei Guo, Peipei Yang, Xue Hu, Juhong Rao, Entao Li, Tong Chen, Sandra Chiu, Gary Wong, Zhiming Yuan, Jiaming Lan, Chao Shan

**Affiliations:** 1 State Key Laboratory of Virology, Wuhan Institute of Virology, Chinese Academy of Sciences, Wuhan, 430071 China; 2 University of the Chinese Academy of Sciences, Beijing, 100039 China; 3 Center for Biosafety Mega-Science, Wuhan Institute of Virology, Chinese Academy of Sciences, Wuhan, 430071 China; 4 Division of Life Sciences and Medicine, University of Science and Technology of China, Hefei, 230026 China; 5 CAS Key Laboratory of Molecular Virology & Immunology, Shanghai Institute of Immunity and Infection, Chinese Academy of Sciences, Shanghai, 200031 China; 6 Hubei Jiangxia Laboratory, Wuhan, 430200 China

**Keywords:** DNA vaccines, Conjugate vaccines

## Abstract

Nipah virus (NiV) is a highly lethal zoonotic paramyxovirus that poses a severe threat to humans due to its high morbidity and the lack of viable countermeasures. Vaccines are the most crucial defense against NiV infections. Here, a recombinant chimpanzee adenovirus-based vaccine (AdC68-G) and a DNA vaccine (DNA-G) were developed by expressing the codon-optimized full-length glycoprotein (G) of NiV. Strong and sustained neutralizing antibody production, accompanied by an effective T-cell response, was induced in BALB/c mice by intranasal or intramuscular administration of one or two doses of AdC68-G, as well as by priming with DNA-G and boosting with intramuscularly administered AdC68-G. Importantly, the neutralizing antibody titers were maintained for up to 68 weeks in the mice that received intramuscularly administered AdC68-G and the prime DNA-G/boost AdC68-G regimen, without a significant decline. Additionally, Syrian golden hamsters immunized with AdC68-G and DNA-G via homologous or heterologous prime/boost immunization were completely protected against a lethal NiV virus challenge, without any apparent weight loss, clinical signs, or pathological tissue damage. There was a significant reduction in but not a complete absence of the viral load and number of infectious particles in the lungs and spleen tissue following NiV challenge. These findings suggest that the AdC68-G and DNA-G vaccines against NiV infection are promising candidates for further development.

## Introduction

Nipah virus (NiV), a member of the *Henipavirus* genus in the *Paramyxoviridae* family, is an enveloped, negative-sense, single-stranded RNA virus. The first recorded cases of NiV occurred in 1998 during an outbreak of severe encephalitis in pig farmers from Malaysia and abattoir workers in Singapore, with a 39.6% case fatality rate (CFR)^1–3^. It was later discovered that pigs were most likely infected after eating fruit contaminated by infected fruit bats of the genus *Pteropus*^4,5^. Humans can become infected with NiV through exposure to sick pigs, but there have been no documented cases of human-to-human transmission^6^. Since the initial outbreak in Malaysia, NiV outbreaks have occurred almost annually in Bangladesh and India between 2001 and 2018^7,8^. NiV can spread directly from bats to humans through the consumption of contaminated raw date palm sap in Bangladesh^9^, with a higher fatality rate ranging from 40% to 75%^10^, and human-to-human transmission has been observed in these outbreaks^11,12^. The latest outbreaks occurred in 2021 in Kerala, India, in which a 12-year-old boy died due to NiV infection^13^. Based on zoonotic spillover events and genetic analysis, at least two distinct major strains, NiV-Malaysia (NiV-M) and NiV-Bangladesh (NiV-B), have been implicated in previous outbreaks in humans^14,15^ Although there are only approximately 650 cases of NiV infection in these outbreaks, the mortality rate can be as high as 100% depending on the context and limitations of the health-care systems in which outbreaks occur.

The symptoms of NiV disease (NVD) include fever, headache, cough, sore throat, difficulty breathing, and vomiting. The illness can progress to more severe symptoms, such as disorientation, drowsiness, acute respiratory illness, and fatal encephalitis^16,17^. Most people who survive acute encephalitis fully recover, but long-term side effects of Nipah virus infection have been reported in survivors, including persistent convulsions and personality changes^10,18^.

Unlike other paramyxoviruses, NiV exhibits a broad host range, including humans, pigs, dogs, cats, horses, guinea pigs, hamsters, and fruit bats^19,20^. NiV uses the highly conserved ephrinB2 and ephrinB3 as host cell entry receptors, which explains its preferential tropism for the lungs, brain, and spleen, all of which express high levels of these cellular receptors^21–23^.

NiV is a risk group 4 pathogen due to its pandemic potential, extreme pathogenicity, and lack of medical treatments. It is listed as a priority pathogen by the World Health Organization (WHO)^24^, but currently, no licensed vaccine or antiviral drug is available. The current NiV vaccine development platform is based on NiV glycoprotein (G) and/or fusion protein (F) and aims to induce the production of NiV neutralizing antibodies. Multiple vaccine approaches have been tested in animal models and have been shown to be effective, such as a recombinant adeno-associated virus vaccine expressing NiV-M G completely protecting hamsters against the NiV-M challenge^25^, a recombinant VSV-vectored vaccine expressing NiV-B G protecting African green monkeys against a homologous NiV-B challenge^26^, and a canarypox virus-based vaccine vector expressing NiV-M G or F protecting against NiV-M in pigs^27^. In addition, a messenger RNA (mRNA-1215) vaccine encoding NiV-M F and G was tested in phase I clinical trials in 2022^28^. Additionally, researchers are also exploring other vaccine platforms to develop novel NiV vaccines with better immune protection effects.

Several vaccines based on AdC68 vectors, including the Ebola virus (EBOV), Middle East respiratory syndrome coronavirus (MERS-CoV), and severe acute respiratory syndrome coronavirus 2 (SARS‑CoV‑2) vaccines, have been developed due to low pre-existing immunity in humans^29^, their broad tissue tropism, their inability to replicate after immunization, and their ability to induce strong and long-lasting immunity^30–32^. In addition, DNA-based vaccines have several advantages, including rapid production, easy design, low temperature sensitivity, and affordability, which are critical elements for outbreak areas. Here, we developed two vaccines, a DNA vaccine (DNA-G) and a recombinant chimpanzee adenovirus vaccine (AdC68-G), both of which express NiV-G, and their immunogenicity and efficacy were tested in mice and hamsters. The results show that the two vaccines induced the production of long-lasting neutralizing antibodies and robust T-cell responses against the G protein after vaccination in BALB/c mice, as well protection from lethal NiV-M and NiV-B infection challenge in Syrian golden hamsters.

## Results

### AdC68-G and DNA-G were successfully constructed and characterized

**Fig. 1:**
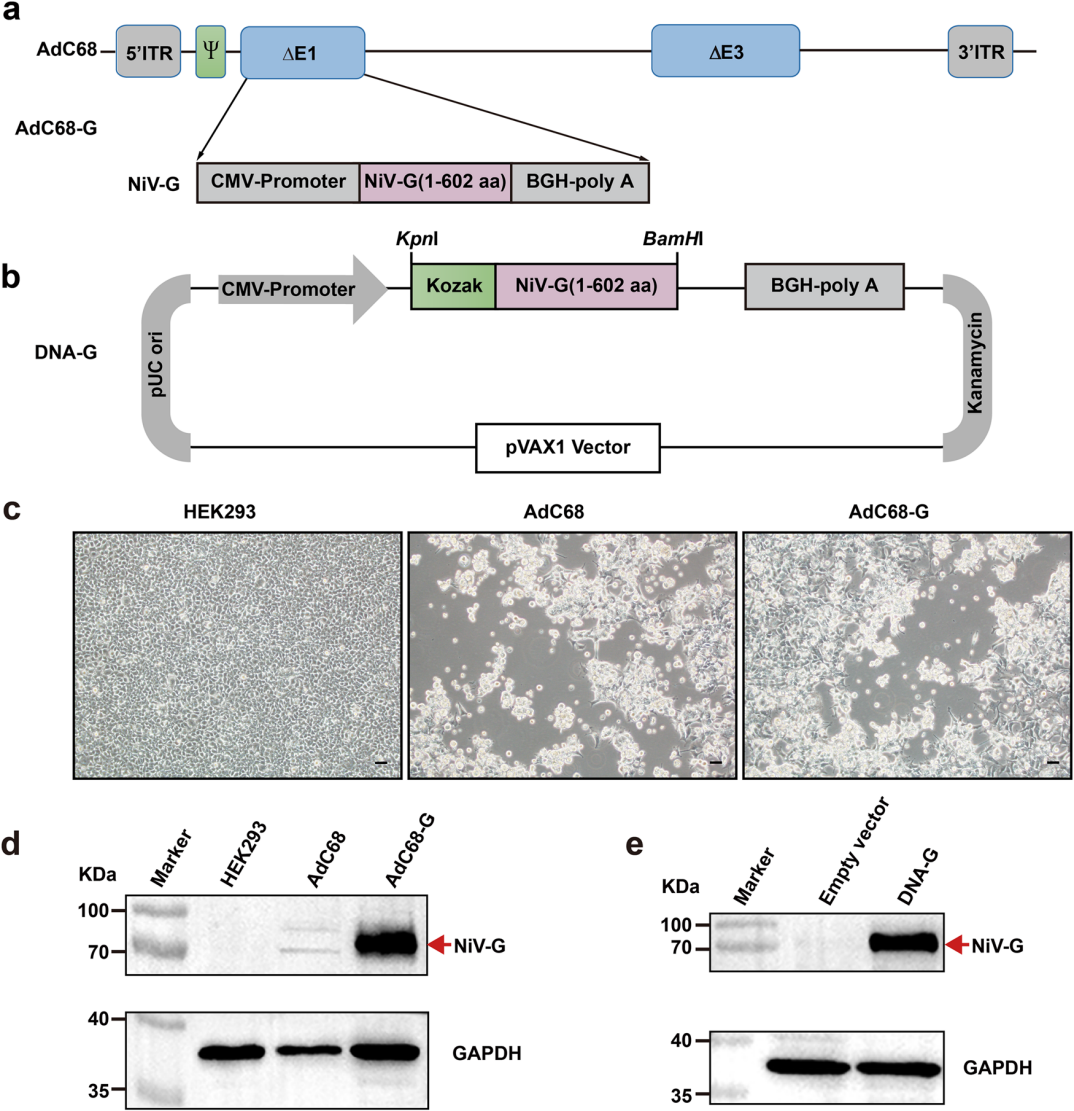
Construction and characterization of AdC68-G and DNA-G. **a** Schematic diagram of the genome of AdC68-G and the coding sequence for the NiV G protein. **b** Diagram of plasmid DNA-G. The plasmid pVAX1 vector was used to engineer a gene cassette containing a CMV promoter, the Kozak sequence, NiV-G cDNA, and a BGH pA signal element. **c** The cytopathic effect (CPE) was observed in HEK293 cells transfected with the linearized AdC68-G or AdC68 plasmid. **d** Western blot analysis of HEK293 cells infected with rescued recombinant AdC68-G or AdC68 viruses. **e** Western blot analysis of HEK293T cells transfected with DNA-G (pVAX1-G) or empty vector (pVAX1). GAPDH was used as the internal control. All blots derive from the same experiment and that they were processed in parallel. Bar scale, 100 µm.

The consensus G protein of NiV shown in Supplementary Fig. 1a was selected as the antigen for vaccine development. The results of phylogenetic tree analysis among the sequence in the study and other unduplicated and complete G protein sequences in GenBank were shown in Supplementary Fig. 1b. The acquired G amino acid sequence in the study has homology with the challenged Malaysian and Bangladesh NiV strains of 96.2% and 99.5% respectively. To develop a recombinant chimpanzee adenovirus-based vaccine (AdC68-G), the full-length G sequence was inserted into the E1-deleted region (E1) of the adenoviral vector (AdC68) and placed under the control of the cytomegalovirus (CMV) promoter (Fig. 1a). In addition, the G gene was subcloned into the mammalian expression vector pVAX1 to generate DNA-G (Fig. 1b). Transfection of HEK293 cells with the linearized recombinant plasmid pAdC68-G resulted in cytopathic effects 8–10 days later (Fig. 1c). The expression of the G protein was evaluated by Western blot analysis of HEK293 cells infected with AdC68-G and transfected with DNA-G using mouse anti-NiV G serum. The results showed high expression of the G protein by AdC68-G and DNA-G, while no G-specific band was detected in the control group (Fig. 1d, e), demonstrating that both a recombinant chimpanzee adenovirus-based vaccine and a DNA-based vaccine that express the full-length codon-optimized NiV G protein were successfully constructed.

### Immunization with AdC68-G alone or in combination with DNA-G vaccine induce robust cellular robust cellular and sustained humoral immune responses in BALB/c mice

**Fig. 2:**
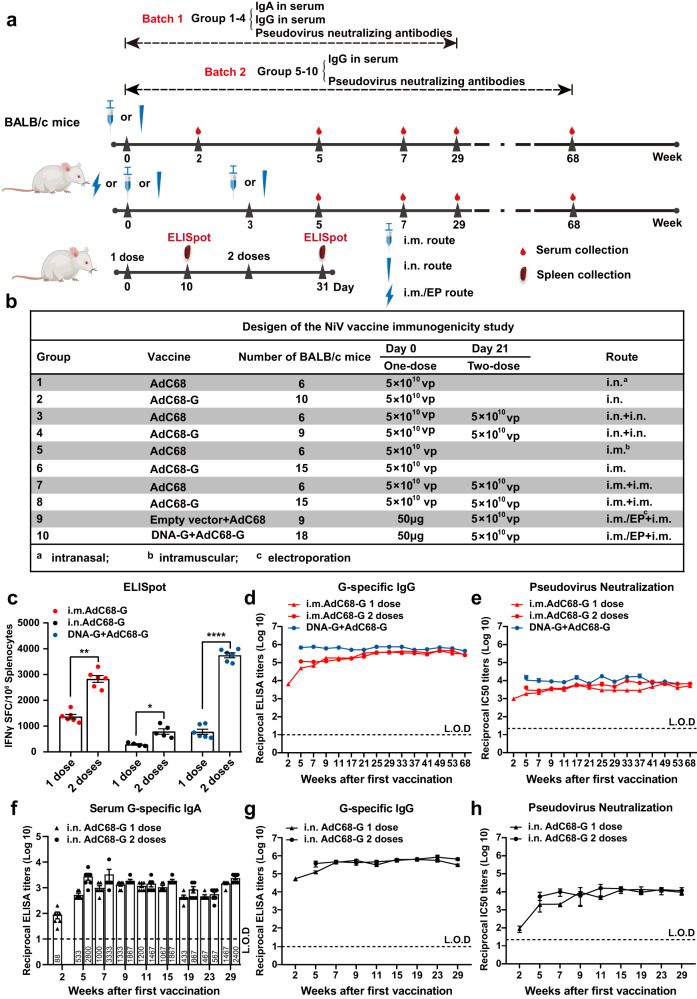
The immunogenicity of AdC68-G and DNA-G in BALB/c mice. Experimental scheme. BALB/c mice were immunized with a recombinant vaccine (AdC68-G or DNA-G) either once (1 dose) or twice (2 doses) at a 21-day interval (**a**) with the indicated schedule, dosage, and route (**b**). Ten days after each immunization, splenocytes were harvested for NiV-specific T-cell response analysis (**a**, lower). **c** T-cell responses induced by the vaccine regimens. IFN-γ-secreting splenocytes induced by the vaccine in BALB/c mice were detected by ELISpot at 10 days after the first or second immunization. Splenic lymphocytes were stimulated with two peptide pools corresponding to the NiV-G region. The IgG antibody (**d**) and pseudovirus neutralization antibody (**e**) responses in the sera of mice immunized with AdC68-G either once (1 dose) or twice (2 doses) via the i.m. route and the DNA-G prime/AdC68-G boost immunization regimen. The IgA antibodies (**f**), IgG antibodies (**g**) and pseudovirus neutralization antibodies (**h**) in the sera of mice immunized with AdC68-G either once (1 dose) or twice (2 doses) via the i.n. route. Data are shown as the mean ± SEM. Two-tailed unpaired Student’s *t* tests were conducted to compare differences between two experimental groups *p < 0.05, **p < 0.01, ****p < 0.0001. Error bars indicate 95% confidence intervals. L.O.D. represents the limit of detection.

To evaluate the immunogenicity of the two vaccines, female BALB/c mice aged 6-8 weeks in ten groups were immunized in two batches (Fig. 2a). The first batch (groups 1 to 4) and the second batch (groups 5–10) were followed for 29 and 68 weeks, respectively. In groups 2 and 4, 5 × 10^10^ viral particles (VPs) of AdC68-G were administered to mice in one dose or two doses at a 21-day interval via the intranasal (i.n.) route, while mice in groups 1 and 3 received 5 × 10^10^ VPs of AdC68-empty vector and were used as controls. In groups 6 and 8, 5 × 10^10^ VPs of AdC68-G was administered to mice in one dose or two doses at a 21-day interval via intramuscular (i.m.) injection, while mice in groups 5 and 7 received 5 × 10^10^ VPs of AdC68 vector and were used as controls. Mice in group 10 received a heterologous DNA-G (50 μg) prime/AdC68-G (5 × 10^10^ VPs) boost vaccine regimen with a 3-week interval, while mice in group 9 were given the corresponding empty vector as a control (Fig. 2b).

To detect NiV-specific T-cell responses, splenocytes were harvested for analysis of the ability to produce interferon (IFN)-γ 10 days after vaccination. The results showed that a strong cellular immune response was induced after one dose of vaccine, while a higher level of IFN-γ produced by T cells was detected after two doses of vaccine (*p* < 0.01, *p* < 0.05 and *p* < 0.0001 in the three different groups). After two doses, the DNA-G prime/AdC68-G boost vaccine group exhibited the highest level of IFN-γ among all regimen groups, with an average of 3746 spot-forming units (SFU) per 10^6^ splenocytes (Fig. 2c).

Serum samples were collected up to 68 weeks post-vaccination in groups 5 to 10, and humoral immune responses with the vaccine regimens were evaluated by enzyme-linked immunosorbent assays (ELISA) and pseudovirus neutralization assay using an HIV-luciferase system. A single i.m. injection of the AdC68-G vaccine was able to induce IgG antibody titers of 10^5^. After homologous boosting with the AdC68-G vaccine, IgG antibody titers were slightly elevated (*p* < 0.01). Heterologous immunization with the DNA-G prime/AdC68-G boost regimen achieved the highest IgG antibody titers, with an average titer of 443733 at 68 weeks after two doses (*p* < 0.0001). Notably, the serum IgG antibody levels of mice in all immunized groups did not decrease significantly until 68 weeks after immunization (Fig. 2d). In accordance with the IgG antibody results, neutralization antibody titers exhibited the same results. The highest average IC_50_ of pseudovirus-neutralizing antibodies (pNAbs) in 68-week immune sera was 6729 for the DNA-G prime/AdC68-G boost immunization group (Fig. 2e).

The humoral immune responses induced by AdC68-G vaccine via i.n. immunization was investigated in mice. G-specific IgA and IgG antibodies were detected as early as two weeks after the first vaccination, and their titers increased upon boosting with AdC68-G (*p* < 0.01) (Fig. 2f, g). In addition, pNAbs whose production was induced by i.n immunization were more potent than those induced by i.m. immunization (Fig. 2g, h). The average IC_50_ of pNAbs in 29-week immune sera was 9303 for the single-dose AdC68-G (i.n.) group and 11,570 for the two-dose AdC68-G (i.n.) group (Fig. 2h).

These findings suggested that all vaccine regimens elicited effective cellular and long-term humoral immune responses in BALB/c mice. However, the two-dose immunization groups exhibited stronger cellular and higher-level humoral immune responses than the single-dose groups. I.n. immunization with AdC68-G induced a stronger humoral immune response than i.m. immunization, and the heterologous DNA-G prime/AdC68-G boost regimen induced a higher humoral and cellular immune response than the homologous AdC68-G/AdC68-G i.m. immunization protocols.

### AdC68-G and DNA-G vaccines elicited the production of virus-neutralizing antibodies against two different strains of NiV in Syrian golden hamsters

**Fig. 3:**
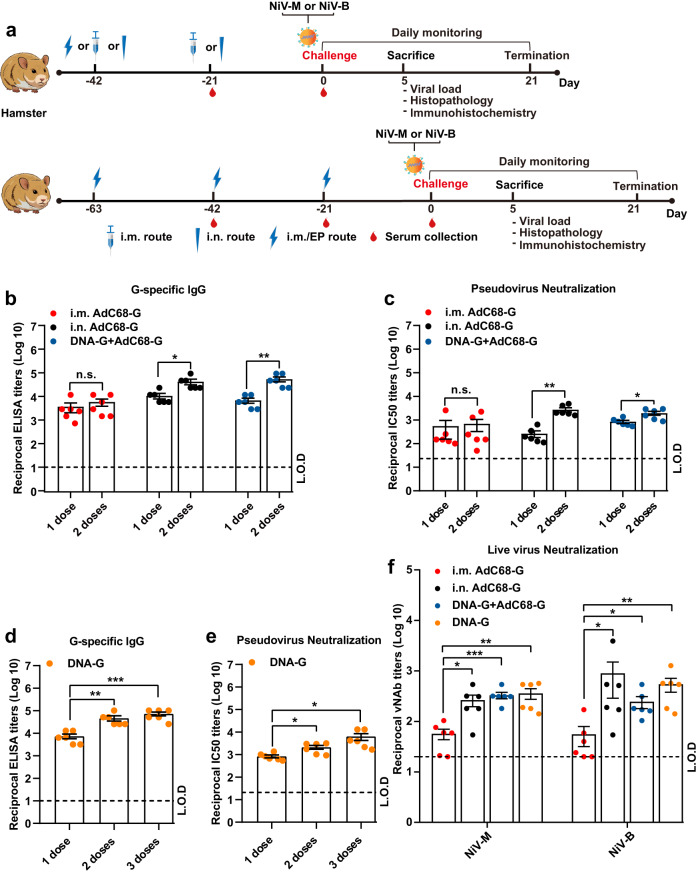
Immunogenicity and challenge schedule in hamsters. **a** The experimental scheme of Syrian hamsters. The animals were vaccinated at day 0 with AdC68-G (via the i.n. or i.m. route) or DNA-G (i.m./EP) and boosted with AdC68-G (via the i.n. or i.m. route) after 21 days (the upper) or immunized 3 times with DNA-G via the i.m./EP route (the lower) and then challenged with the NiV Malaysia and Bangladesh strains at 1000 LD_50_ via the i.p. route. Hamsters were monitored daily for survival as well as body weight changes. Six hamsters in each group (except for those in the AdC68-G (i.n.) group that was challenged with NiV-M, *n* = 3) were euthanized at 5 d.p.i. for viral load and pathogenesis analysis. The IgG antibodies (**b**) and pNAbs (**c**) on day 21 after the first or second vaccination. The IgG antibodies (**d**) and pNAbs (**e**) on day 21 after the first, second and third vaccinations. **f** vNAb detection based on the live NiV Malaysia and Bangladesh strains at 3 weeks after the last immunization. Data are presented as the group mean ± SEM. Statistical significance was determined one-way ANOVA with Tukey’s multiple comparisons tests. n.s., not significant, *p < 0.05, **p < 0.01, ***p < 0.001. Error bars indicate 95% confidence intervals. L.O.D. represents the limit of detection.

Before determining whether the AdC68-G and DNA-G vaccines provide protection against NiV infection, the immune response induced by these vaccine regimens was evaluated in hamsters. Based on the immunogenicity of vaccination in BALB/c mice, a two-dose regimen was chosen for this study, with the addition of a three-dose DNA-G vaccine regimen. The vaccination schedule of the study is summarized in Fig. 3a. Briefly, hamsters were vaccinated on day 0 with AdC68-G (i.n. or i.m.) or DNA-G intramuscularly and electroporation (i.m./EP) and boosted with one dose of AdC68-G (i.n. or i.m.) or two doses of DNA-G (i.m./EP) at 21-day intervals. As a negative control, hamsters were vaccinated with empty AdC68 or pVAX1 vectors at the same time. Blood samples were collected at 3 weeks after each vaccination. High levels of G-specific IgG and pNAbs were detected after a single dose.

Regarding the 2-dose groups, the results showed that after the first immunization, the AdC68-G and DNA-G regimens induced high levels of G-specific IgG and neutralizing antibodies, and the second vaccination resulted in a statistically significant increase (*p* < 0.05) in IgG (Fig. 3b) and pNAb (Fig. 3c) titers, except for in the AdC68-G (i.m.) group. For the 3-dose DNA-G vaccine group, G-specific IgG (Fig. 3d) and pNAbs (Fig. 3e) were detected in all hamsters, and pNAb titers increased (*p* < 0.05) following each immunization (Fig. 3e).

Hamster sera from both the AdC68-G and DNA vaccine regimen groups could neutralize two strains of NiV (Malaysia and Bangladesh), with the highest virus-neutralizing antibody (vNAb) titers of 567 (NiV-M) and 3903 (NiV-B), respectively. The vNAb titers in hamsters immunized with AdC68-G via the i.m. route was significantly lower (*p* < 0.05) than those in the hamsters in the other 3 groups, with two of the six animals in the AdC68-G (i.m.) group not having detectable neutralizing antibodies (vNAb titers <20). There was no significant difference in the neutralizing abilities against the NiV Malaysia and Bangladesh strains among two-dose AdC68-G (i.n.), DNA-G prime/AdC68-G boost and DNA-vaccinated animals (Fig. 3f). No IgG or neutralizing antibodies were detected in any of the control animals. These results indicated that the AdC68-G and DNA-G vaccines induced a potent humoral immune response in Syrian golden hamsters.

### AdC68-G and DNA-G vaccines completely protected Syrian golden hamsters from lethal NiV infection

**Fig. 4:**
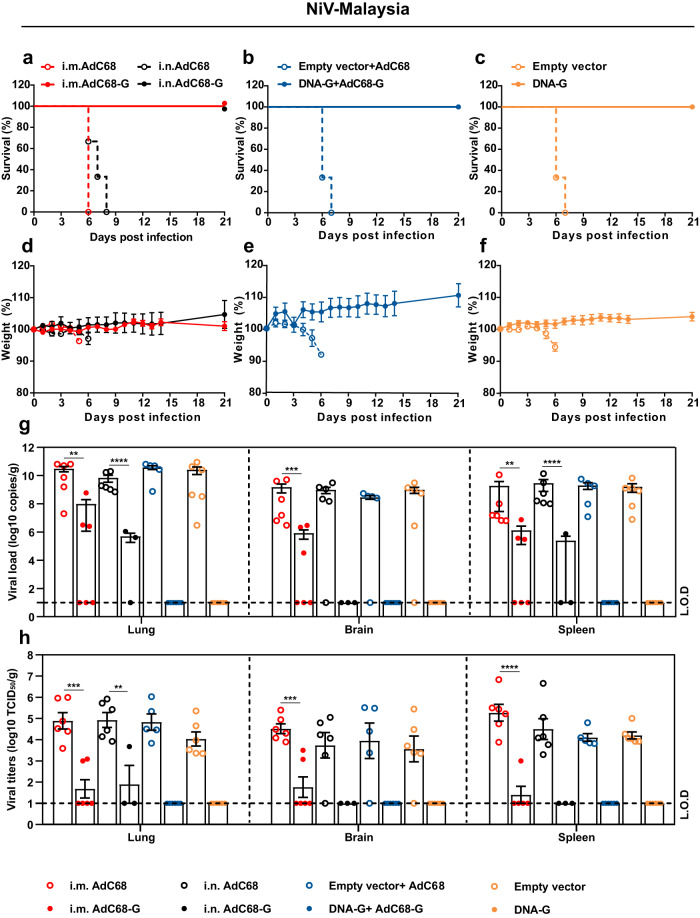

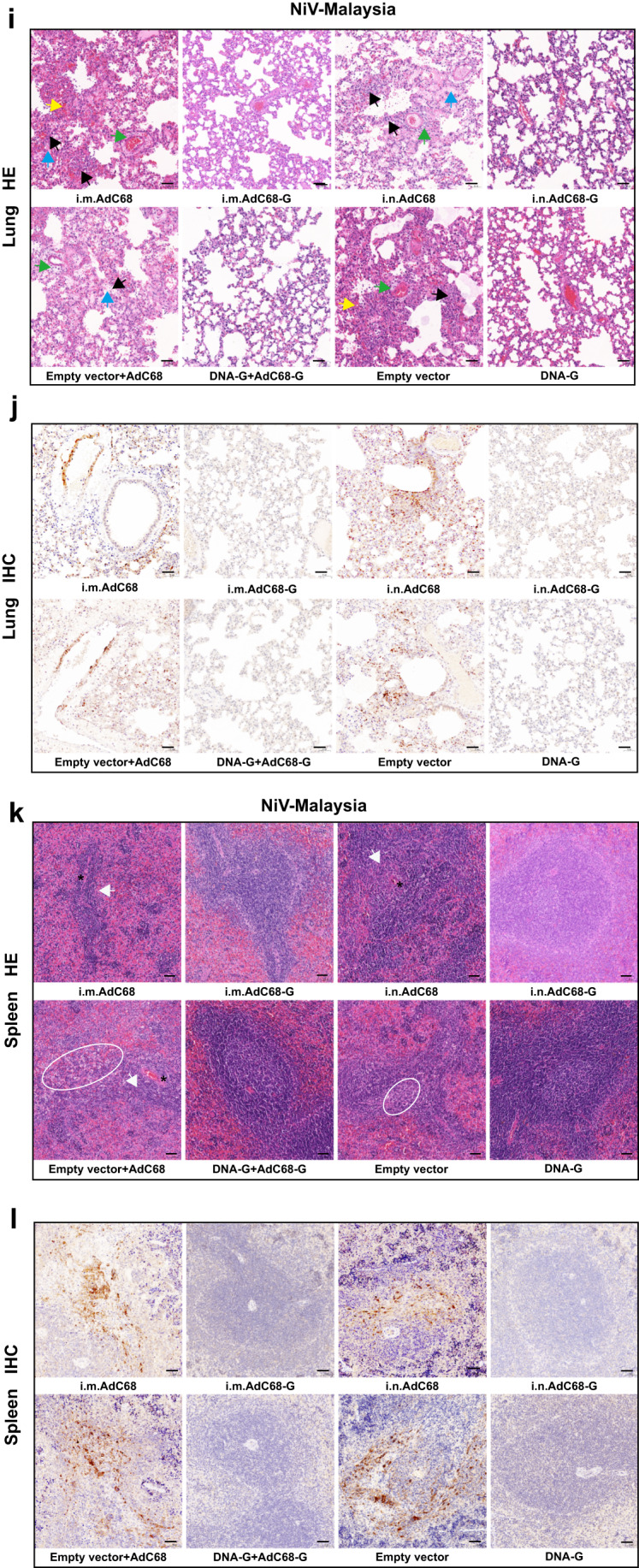
AdC68-G and DNA vaccines protected Syrian golden hamsters from lethal NiV Malaysia challenge. Survival (**a**–**c**) and weight change (**d**–**f**) of Syrian hamsters challenged with the NiV Malaysia strain. Viral loads in the hamster lungs, brain, and spleen at 5 d.p.i. quantified by quantitative PCR with reverse transcription (qRT–PCR) (**g**) and live virus titration (**h**). **i** Lung tissue sections were stained with hematoxylin and eosin (HE). Marked bronchointerstitial pneumonia with inflammatory cell infiltration or necrosis (black arrows), hemorrhage (yellow arrows), vasculitis (green arrows), and fibrous exudation (blue arrows) in the lung tissue of control animals. No pathology was observed in vaccinated animals. **j** Lung tissue sections were stained with an antibody against the NiV N antigen, which was visible as red‒brown staining (IHC). No immunoreactivity was found in vaccinated animals, whereas multifocal immunoreactivity could be found in the lung tissue of control animals. **k** Spleen tissue sections were stained with hematoxylin and eosin (HE). Loss of normal splenic architecture, with lymphocyte necrosis (white circles) and decreased lymphocyte numbers in white pulp (white arrows); the asterisk indicates the central artery. **l** Spleen tissue sections were stained with an antibody against the NiV N antigen, and red‒brown color indicates immunolabeled mononuclear or multinucleated cells. Data are presented as the group mean ± SEM. Two-tailed unpaired Student’s *t* tests were conducted to compare differences between two experimental groups. **p < 0.01, ***p < 0.001, ****p < 0.0001. Error bars indicate 95% confidence intervals. L.O.D. represents the limit of detection. Bar scale, 50 µm.

To confirm whether the vaccine regimen-induced immune response could provide protection against NiV challenge, all vaccine-immunized hamsters were challenged with a lethal dose of NiV (1000 LD_50_) via the intraperitoneal (i.p.) route 3 weeks after the last immunization (Fig. 3a). Hamsters (*n* = 3 per control group, *n* = 6 per vaccine group) were monitored daily for survival and body weight changes over a 21-day period, and six hamsters in each group were sacrificed at 5 days post-infection (d.p.i.) for viral load determination as well as pathogenesis analysis. During the monitoring stage, all the hamsters in the vaccine groups survived the lethal challenge, with no obvious signs of disease, and maintained a relatively stable body weight after NiV-M (Fig. 4a–f) and NiV-B infection (Supplementary Fig. 2a–f). In contrast, animals in the control group succumbed to the infection between 6 and 8 d.p.i. for NiV-M infection (Fig. 4a–c) and between 6 and 14 d.p.i. for NiV-B infection. Unfortunately, one hamster survived NiV-B infection in each control group, including the AdC68 (i.m. or i.n.) and pVAX1 prime/AdC68 boost groups, but there was a significant reduction in body weight (*P* < 0.05) even though euthanasia was not necessary (Supplementary Fig. 2a, b, d, e). The hallmark symptoms of disease, such as ruffled fur, slowed activity, hunched posture, bleeding, and labored breathing, were observed in the animals of the control group. The vaccine regimens completely prevented these symptoms. The clinical scores of the challenged hamsters were recorded (Supplementary Fig. 3). Statistical analysis demonstrated that survival in all vaccinated groups was significantly better than that in the corresponding control groups (*p* < 0.05).

Next, six hamsters in each group were euthanized at 5 d.p.i. to determine the viral load as well as the pathological changes in the lungs, brain and spleen. NiV RNA could not be detected in any of the three organs of the animals in the DNA-G and DNA-G prime/AdC68-G boost vaccine groups after NiV-M (Fig. 4g) or NiV-B (Supplementary Fig. 2g) challenge. For individual animals immunized with AdC68-G via the i.m. or i.n. route, NiV RNA was detectable, but the copy number of viral RNA was significantly lower (*p* < 0.01) in the tissues than those in the control group after challenge with NiV-M (Fig. 4g). The infectious particles were also examined in the tissues and showed the same pattern as viral RNA (Fig. 4h). In the NiV-B challenge experiment, all the animals in the vaccine groups except for those that received the AdC68-G vaccine via the i.m. route vaccination showed complete viral RNA elimination (Supplementary Fig. 2g). Infectious particles could not be detected in any tissues in any of the vaccine-immunized animals (Supplementary Fig. 2h). Pathological analysis of lung and spleen tissue collected at 5 d.p.i. revealed that the vaccinated hamsters maintained normal tissue structures with no obvious pathological damage after NiV-M (Fig. 4i, k) or NiV-B infection (Supplementary Fig. 2i, k), and no viral antigen was detected in lung and spleen tissues by immunohistochemistry (IHC) labeling for NiV nucleoprotein (N) antigen after NiV-M (Fig. 4j, l) and NiV-B (Supplementary Fig. 2j) infection. In the control group, decreases in lymphocyte levels and the loss of normal structures were observed in the spleen. Alveolar spaces had hemorrhage, edema and fibrin deposition with inflammatory cells, and inflammation often surrounded vascular and alveolar walls, consistent with a previous report (Fig. 4i, k; Supplementary Fig. 2i, k). In addition, NiV N antigen was detected by IHC in mononuclear cells, multinucleated cells, and the endothelium within the spleen and lungs (Fig. 4j, l; Supplementary Fig. 2j). Thus, these results indicated that the vaccines almost completely abolished viral replication and significantly alleviated the severity of lung and spleen pathology, ultimately providing complete protection against lethal NiV challenge.

## Discussion

NiV is a re-emerging zoonotic pathogen that continues to pose a threat to human populations. Due to its high lethality, its potential for spread and the lack of effective medical countermeasures, the WHO has designated NiV as a priority pathogen requiring further research. Several NiV vaccine candidates have been tested in preclinical trials, including nucleic acid vaccines (DNA and mRNA)^28,33,34^, viral vector vaccines^26,35–39^ and subunit vaccines^40–42^. These vaccine candidates yielded different levels of protection against NiV infection; however, no licensed vaccines are available for NiV. Therefore, it is necessary to develop additional novel effective NiV vaccines to induce a potent and long-lasting antibody response and protect against a wide range of strains.

Genotypic analysis divides NiV into two strains: NiV-Malaysia (NiV-M) and NiV-Bangladesh (NiV-B)^14,15^. In our study, we aimed to optimize the immunogenic G sequence and develop a vaccine that protects against both strains using replication-deficient chimpanzee adenoviruses and DNA vaccines expressing full-length G protein. The results showed that a prime-boost approach using either AdC68-G or DNA-G induced a robust and sustained neutralizing antibody and cellular immune response in mice. Although the neutralizing activity of mice sera was detected by pseudovirus neutralization assay based on HIV backbone, the results were convincing, based on the results of others study^33^ and the results of the preliminary experiment (the correlation of live NiV neutralization and pseudovirus neutralization assays) of this study (Supplementary Fig. 4).

The heterologous DNA-G/AdC68-G immunization regimen produced higher T-cell responses and neutralizing antibody titers than homologous AdC68-G immunization. Furthermore, due to the coronavirus disease 2019 (COVID-19) pandemic, the heterologous severe acute respiratory syndrome coronavirus 2 (SARS-CoV-2) vaccine combination is gaining support for improving immunogenicity compared to that with the homologous vaccine administration used in mass vaccination campaigns for humans^43,44^. Though the AdC68-G vaccine of the study is a replication-deficient virus, it can still induce a durable (68 weeks) immune response in mice, which is consistent with other study^30^. While the exact mechanism behind the long-lasting and sustained immune responses is currently unknown^31^. The development of vaccines that induce a lasting immune response against respiratory viruses is currently the most challenging study. To fight against COVID-19, the vaccinees were recommended to be vaccinated every six months. Similarly, the seasonal influenza virus vaccine needs to be administered annually. All these are related to the difficulty of inducing durable immune responses in large animal models by the vaccines. In this study, the AdC68-G vaccine developed against Nipah virus induced an immune response lasting up to 68 weeks without a downtrend, which provides a promising and long-lasting vaccine against respiratory virus infection after being verified in large animal models such as African green monkey.

In human clinical trials, DNA vaccines for a variety of infectious pathogens yielded disappointing results^45^. There are lots of reasons for this poor performance, but a major reason is thought to be insufficient antigenic load due to inefficient expression of the target antigenic proteins in immunologically relevant cells and tissues^46^. To overcome the inefficient expression of the target antigen, EP is one of the most effective solutions. Histological analysis of the site of injection revealed that the transfection efficiency was greatly improved and the variability between animals was reduced when the DNA vaccines were delivered by EP. The advantage of in vivo EP is that it does work in larger animals, including humans^47^. Based on these, the delivery of the DNA vaccine in this study was performed by i.m/EP. Consistent with another research study^48^, the humoral and cellular immune responses induced by the DNA vaccine (delivered by EP) are not lower than or even higher than those of the protein vaccine under adjuvant.

Several immunization regimens with both vaccines provide complete protection against NiV infection. The heterologous DNA-G/AdC68-G and three-dose DNA immunization regimens were found to be superior to the homologous AdC68-G regimen. In the current study, we used the standard three-dose immunization regimen for the DNA vaccine. However, our immunogenicity data clearly show that a two-dose immunization regimen can induce a robust humoral immune response, and whether it can fully protect against NiV infection needs to be investigated further.

For the lethal NiV-B challenge, the infectious particles were eliminated in all vaccinated animals, while after the NiV-M challenge, no or reduced numbers of infectious particles were detected in the lungs, brain, and spleen in the i.n. and i.m. vaccinated groups. It is possible that our optimized G amino acid sequence had higher homology to that of NiV-B than to that of NiV-M. The Bangladesh strain shares 99.3% identity, while the Malaysia strain shares 96% identity. Notably, animals were given a high dose of the virus (1000 LD_50_) for the challenge in the current model. This infection dose is probably higher than that in a natural infection in humans.

In an outbreak scenario, an ideal vaccine should have the ability to elicit protective immunity after a single immunization dose. According to previous research, the developed adenovirus vector vaccine is capable of protecting against a virus challenge after a single immunization dose^32,49^. Thus, we investigated whether a single dose of AdC68-G (delivered by the i.m. or i.n. route) was sufficient to elicit an effective immune response in this study. The data presented here show that the prime-only approach induced robust T-cell responses and high levels of long-lasting neutralizing antibodies in BALB/c mice. The i.m. administration of AdC68-G elicited a significantly higher level of IFN-γ than i.n. AdC68-G administration. However, i.n. AdC68-G immunization appears to be more effective than i.m. immunization at inducing serum IgA immune responses. Whether the induced IgA can play the role of antibody-dependent cell-mediated cytotoxicity and then destroy the target cells infected by the NiV needs further study. Based on the results presented here, further studies are planned to test whether a single vaccination with AdC68-G can also protect animals against NiV challenge.

In conclusion, our results suggest that both homologous AdC68-G (delivered by i.m. or i.n. administration), heterologous DNA-G/AdC68-G prime/boost, and DNA-G three-dose approaches are effective in inducing immunity against Nipah virus infection in animal models. The heterologous DNA-G/AdC68-G prime/boost approach induced higher T-cell responses and neutralizing antibody levels, while a single dose of AdC68-G was sufficient to elicit a strong immune response in mice. Further studies are needed to determine if these findings can be applied to humans.

## Methods

### Ethics statement

The animal experiments were approved by the Animal Ethics Committee of the Wuhan Institute of Virology, Chinese Academy of Sciences (approval number: WIVA21202104). All animal experiments involving NiV were executed in the animal biosafety level 4 (ABSL-4) facility at the National Biosafety Laboratory (Wuhan), Chinese Academy of Sciences.

### Cells and virus

HEK293T (ATCC: ACS-4500), HEK293 (ATCC: CRL-1573) and Vero E6 (ATCC: CRL-1586) cells were maintained in Dulbecco’s modified Eagle’s medium (DMEM, Gibco) containing 10% Fetal bovine serum (FBS, Gibco), penicillin (100 units/ml) and streptomycin (100 μg/ml) at 37 °C in 5% CO_2_. 293 Freestyle (293 F) cells were maintained in FreeStyle293 expression medium containing penicillin (100 units/ml) and streptomycin (100 μg/ml) in shaker incubators at 150 rpm, 37 °C, 8% CO_2_. The NiV Malaysia (AF212302.2) and NiV Bangladesh (AY988601.1) strains used in the challenge studies were obtained from the National Virus Resource Center, Wuhan Institute of Virology, Chinese Academy of Sciences. The virus stock was propagated in Vero E6 cells.

### AdC68-Based NiV vaccine production

The full-length G sequences used in this study were obtained by optimizing the NiV G sequences of two different NiV-M and NiV-B strains in the software Geneious using the principle of sequence homology. Specifically, all the sequences of G proteins were downloaded from GenBank and the vaccine consensus sequences were acquired according to the sequence consistency principle. To understand the conservation of the acquired consensus sequence, a phylogenetic tree was constructed by the maximum likelihood method in Mega11. The G sequence was then codon optimized for humans and synthesized by GenScript (Nanjing, China). The codon optimized G gene was cloned into a transgene expression plasmid pShuttle2 between the restriction sites *Not*I and *Kpn*I. Subsequently, after digestion with *I-Ceu*I and *PI-Sce*I restriction enzymes, the whole G expression cassette containing the CMV promoter, G gene, and BGH polyA tail was inserted into the E1-deleted region of the chimpanzee adenoviral vector pAdC68 to generate the recombinant pAdC68-G. The resultant pAdC68-G was linearized by *Pac*I and transfected into HEK293 cells to rescue AdC68-G, Finally, AdC68-G as well as empty control AdC68 were propagated and purified by cesium chloride density gradient centrifugation, titrated, and stored at −80 °C.

### NiV DNA vaccine construction

For the construction of the NiV DNA vaccine (also termed DNA-G), The optimized gene was subcloned into the clinically used vector pVAX1 between the restriction sites *Kpn*I and *BamH*I with the Kozak sequence incorporated at the 5’ end of the genes. The plasmid was transformed into *E. Coli* Top 10 cells for plasmid amplification and purified using an endotoxin-free Plasmid Plus Maxi Kit (Qiagen).

### Western blot analysis

HEK293T cells cultured in 6-well plates were transfected with 2 μg of DNA-G or pVAX1 for 48 h. HEK293 cells were infected with AdC68-G and AdC68 at doses of 10^9^ VPs per well. After transfection or infection, the culture supernatant was discarded, and the cells were harvested and lysed with 150 μl of RIPA lysis buffer containing a protease inhibitor cocktail. The samples were run on a 10% PAGE gel and transferred to a PVDF membrane (Millipore). After that, skim milk (5%) was added to block the membrane for about 1 h, and a mouse antibody against NiV-G serum (prepared by our laboratory) was used to incubate the membrane with a 1:500 dilutions. After washes, a horseradish peroxidase (HRP)-conjugated secondary anti-mice IgG (Proteintech, Cat No. SA00001-1, diluted 1:2000) was used to bind to the primary antibody. Finally, the membranes were developed with an ECL substrate (Thermo Scientific). The expression of GAPDH (Abcam, Cat No. ab8245, diluted 1:5000) was used as a loading control. Uncropped and unprocessed scans of are unloaded in the Supplementary Fig. 5.

### Animal experiments

BALB/c mice (6–8 weeks old, female) and Syrian hamsters (5–6 weeks old, female) were purchased from Vital River Laboratories (Beijing, China). BALB/c mice were randomly allocated into 10 groups (Fig. 2b). In batch 1 (groups 1–4), two groups (groups 1 and 3) of mice were vaccinated (one or two doses) with 5 × 10^10^ VPs AdC68 via the i.n. route and were used as controls. Two groups (groups 2 and 4) of mice were vaccinated (one or two doses) with 5 × 10^10^ VPs AdC68-G by i.n. route. In batch 2 (groups 5–10), three groups (groups 5, 7 and 9) of mice were vaccinated with 5 × 10^10^ VPs AdC68 (i.m.) or 50 μg pVAX1 (i.m./EP) at day 0 and AdC68 (i.m.) at day 21 and were used as controls. Three groups (groups 6, 8 and 10) of mice were vaccinated with 5 × 10^10^ VPs AdC68-G (i.m.) or 50 μg DNA-G (i.m./EP) and AdC68-G (i.m.) at day 21. For DNA vaccine immunization in BALB/c mice, anesthetized mice were injected with 50 μl of solution containing indicated DNA, following instillation, the injection site was subject to electroporation at 100 V constant voltage with 3 pulses at 50 msec/pulse and 1 s intervals between pulses. Electric pulses were delivered by the electric pulse generator (ECM830; BTX, San Diego, CA). At day 10 after each vaccination, mice were sacrificed and splenocytes were harvested for the detection of cellular immune responses. Sera were collected at predetermined time intervals for the detection of humoral immune responses (Fig. 2a). The mice were anesthetized by isoflurane before the needle vaccination and retro-obital bleeding. For all the DNA vaccination, the nosecone connected with vaporizer machine was used to maintain the anesthesia stage. For the euthanasia procedure, the mice were anesthetized by isoflurane followed by the cervical dislocation.

Hamsters were randomly divided into 8 groups. Four groups of animals were homologously vaccinated with 5 × 10^10^ VPs AdC68-G or AdC68 via an i.m. or i.n. route at a 21-day interval. Two groups of animals were prime vaccinated with 100 μg of DNA-G or pVAX1 and boosted with 5 × 10^10^ VPs AdC68-G or AdC68 via i.m. route at a 21-day interval. Two groups of animals were immunized thrice with 100 μg of DNA-G or pVAX1 on days 0, 21, and 42, respectively. For DNA vaccine immunization in hamsters, the injection site was electroporated immediately after instillation (100 μl/per animals) with a 75 V constant voltage with 10 pulses at 50 msec/pulse and 100-msec intervals between pulses. The serum samples were collected at the indicated time points and subjected to immunological assays (Fig. 3a). Three weeks after final immunization, all animals were transferred to the ABSL-4 and challenged with 1000 LD_50_ of NiV Malaysia (8.55 × 10^3^ TCID_50_) or Bangladesh (22.33 × 10^3^ TCID_50_) strains in 500 μl DMEM via the i.p. route. Six animals in each group (except for the AdC68-G (i.n.) group that was challenged with NiV-M, *n* = 3) were euthanized 5 d.p.i. The lungs, brain and spleen were collected and subjected to analyze for virology and histology. The remaining animals (*n* = 3 per control group, *n* = 6 per vaccine group) were monitored over a 21-day period. During the monitored period, the clinical scores of the challenged hamsters were recorded daily^50^. The weight of the animals was recorded over the 21-day period. Hamsters with a weight loss of more than 25% (recorded as dead) or at the end of protocol are euthanized by cervical dislocation under isoflurane anesthesia. All animal studies follow the ARRIVE reporting guidelines^51^.

### NiV G protein expression and purification

The codon-optimized stalk and ectodomain of NiV G (residues Q71-T602) was cloned into the pcDNA3.4 mammalian expression vector using *Not*I and *Xba*I to fuse an N-terminal signal peptide and a C-terminal His-Tag. The constructs were expressed by transfection of 293 F cells with PEI transfection reagent (Sigma) according to the manufacturer’s protocol. Transfected cells were incubated in shaker incubators at 150 rpm, 37 °C and 8% CO_2_. The supernatant was harvested 5 days after transfection and centrifuged at 1000 rpm for 30 min to remove cellular debris. The supernatant was filtered through a 0.45 µm filter before the G protein was purified using Ni Sepharose High Performance histidine-tagged protein purification resin (Cytiva) eluted with 500 mM imidazole in 50 mM Tris and 100 mM NaCl pH 8.0. Pooled fractions were dialyzed overnight into 20 mM Tris and 150 mM NaCl and 10% (v/v) glycerol at pH 8.0.

### ELISA

96-well EIA/RIA plates (Corning) were coated overnight at 4 °C with 20 μg of G protein per plate in coating buffer (Solarbio). All wells were blocked with 100 µl of blocking buffer (5% skim milk in PBS) for 1.5 h at 37 °C. After standard washes and blocks, serum (2x serially diluted, starting at 100x dilution) in 5% skim milk in PBS was incubated at 37 °C for 2 h. Plates were washed four times in PBS with 0.1% Tween 20 (PBST) before addition of HRP-conjugated goat anti-mouse IgG (Abcam, Cat No. ab6789, diluted 1:20,000), IgA (Abcam, Cat No. ab97235, diluted 1:10,000) or HRP-conjugated goat anti- hamster IgG (Abcam, Cat No. ab6892, diluted 1:15,000) diluted in 5% milk in PBS for 1.5 h at 37 °C. Plates were washed as before prior to being developed with 100 µl/well of TMB chromogen solution (Beyotime) for 15 min. Substrate reactions were stopped by the addition of 50 µl/well of stop solution for TMB Substrate (Beyotime) before reading plate absorbance at 450 nm (OD450). The cutoff value was defined as 2.1-fold of OD450 values from the sample of nonvaccinated mice. The reciprocal of the maximum sample dilution with OD450 values equal to or greater than the cutoff value was used to calculate the endpoint binding antibody titers.

### ELISpot assays

An ELISpot assay for the detection of IFN-γ-secreting mouse splenocytes was performed with a mouse IFN-γ ELISpot kit (Mabtech). Feshly harvested splenocytes of 5 × 10^5^ per well incubated with pools of NiV G peptides (18-mers with 10 amino acid overlap). The final concentration of each peptide was 2 μg/ml. The cells were then incubated with 5% CO2 at 37 °C. 24 h later, IFN-γ spot-forming cells were detected by staining membranes with anti-mouse IFN-γ-biotin (1 μg/ml) followed by streptavidin-ALP. Phorbol 12-myristate 13-acetate and 10 μg/ml ionomycin (Dakewe) was added to the positive-control group, whereas the negative-control group received no stimuli. Analysis was performed using the CTL ImmunoSpot Analyzer and ImmunoSpot Software (Cellular Technology). Spot-forming unit (SFU) per million cells was calculated.

### Neutralization assay based on NiV pseudotyped lentivirus

NiV pseudovirus bearing the F25 (1-523 aa) and G33 (34-602 aa) proteins of NiV was produced in an Env-defective, luciferase-expressing HIV-1 backbone (pNL4-3. Luc-R-E-). Briefly, the F25 and G33 sequences of NiV were synthesized and subcloned into the pcDNA3.1(+) plasmid (GenScript). A total of 8 × 10^6^ HEK293T cells were seeded into a 75 cm^2^ flask and co-transfected with 30 μg of pNL4-3. Luc-R-E- and 1.7 μg of pcDNA3.1( + )-F25 and 3.3 μg of pcDNA3.1( + )-G33 using the Lipofectamine 2000 transfection reagent (Thermo Scientific). 48 h later, the supernatants were filtered and stored below −80 °C. In the serum neutralization assay, samples were heat-inactivated at 56 °C for 30 min, and pseudovirus was diluted and mixed with 3-fold serial dilution of samples in 96-well plates. After 1 h incubation at 37 °C, 3 × 10^4^ of HEK293T cells were seeded on a serum pseudovirus-mixture. 48 h later, the activity of luciferase was measured using a Luciferase Assay System (Promega). Neutralization titers were calculated as the serum dilution at which RLU were reduced by 50% compared with RLU in virus control wells.

### Neutralization assay based on NiV live virus

Three-fold serial dilutions, starting at a 1:20 dilution, of heat-inactivated (30 min, 56 °C) sera were prepared in DMEM containing 2% FBS. The samples were then added to either 100 TCID_50_ NiV-M or 50 TCID_50_ NiV-B, subsequently incubated at 37 °C for 1 h. Following incubation, the virus-serum mixtures were incubated for 1 h with Vero E6 cells, at 37 °C with 5% CO2. The cytopathic effect (CPE) was scored on wells at 5 dpi. The neutralization titer was expressed as the reciprocal of highest dilution of the serum that prevented infection of 50% of quadruplicate inoculations.

### Virus titration assay

Tissue sections were weighed and homogenized in 1 ml of DMEM, Then, 100 μl of 10-fold serial dilutions of tissue homogenate was added to Vero E6 cells in a 96-well plate in quadruplicate. After 1 h incubation at 37 °C and 5% CO_2_, cells were washed with PBS and 100 μl of DMEM containing 2% FBS was added. Cells were incubated at 37 °C and 5% CO_2_. Cytopathogenic effects were assessed 5 days later, and results are expressed as TCID_50_ according to the method of Reed and Muench.

### qRT-PCR

After the hamsters were euthanized, lungs, brain and spleen were collected for viral load examination. The tissues were homogenized in 1 ml DMEM in a tissue grinder. 140 μl of the homogenate supernatant was then subjected to RNA isolation using the Qiagen RNeasy Mini kit (Qiagen), and the total RNA was eluted in 50 μl RNase-free water. Real-time quantitative RT-PCR (qRT-PCR) was performed on a CFX96 Real-Time System (Bio Rad) using the HiScript II One Step qRT-PCR Probe Kit (Vazyme) targeting the NiV nucleocapsid (N) gene. Primers and probes used were: forward primer (5’-AACATCAGCAGGAAGGCAAGA-3’), reverse prime (5’-GCCACTCTGTTCTATAGGTTCTTC-3’), probe (5’-FAM-TTGCTGCAGGAGGTGTGCTC-BHQ1-3’) The standard curve was constructed with nine points in a 20 µl reaction system (10^9^–10^1^ copies). Samples < 10^1^ copies were defined as negative.

### Histology and immunohistochemistry

Lungs, brain and spleen tissue were fixed in 10% formalin for 7 days with two volume change before being transferred out of the BSL-4, following standard operating procedure approved by the Institutional Biosafety Committee. Then the samples were embedded in paraffin, sequentially sectioned to 4 µm thickness, and stained with haematoxylin and eosin (HE) prior to examination by light microscopy. To obtain a representative result, different sites of the lung tissue and the almost whole spleen were sampled for the histopathology analysis.

Specific anti-NiV immunoreactivity was detected using a rabbit anti-NiV N protein antibody (prepared by our laboratory) at 1:3000 dilutions for overnight at 4 °C. The secondary antibody used was biotinylated goat anti-rabbit IgG (SeraCare, Cat No.5220-0336) at 1:500 dilutions for 50 min. Slides were developed with DAB chromogen for 15 s and counterstained with hematoxylin for 45 s. The image was collected by a Pannoramic MIDI system (3DHISTECH, Budapest, Hungary).

### Statistical analysis

The statistical analysis was performed with GraphPad Prism 8.0 software. Two-tailed unpaired Student’s *t* tests were conducted to compare differences between two experimental groups. The One-way ANOVA with Tukey’s multiple comparisons tests were applied to compare more than two experimental groups. *p < 0.05, **p < 0.01, ***p < 0.001, ****p < 0.0001. n.s., not significant.

### Reporting summary

Further information on research design is available in the Nature Research Reporting Summary linked to this article.

### Supplementary information

Reporting Summary

Supplementary information

## Data Availability

The data that support the findings of this study are available from the corresponding author upon reasonable request.
