# Supplementary material for: Both chimpanzee adenovirus-vectored and DNA vaccines induced long-term immunity against Nipah virus infection
Source: NPJ Vaccines. 2023 Nov 4;8:170. doi: 10.1038/s41541-023-00762-3 (PMC10625554; doi:10.1038/s41541-023-00762-3)
Supplement: Supplementary file 1 — Reporting Summary [file 41541_2023_762_MOESM1_ESM.pdf]

nature portfolio

Corresponding author(s): Chao Shan, Jiaming Lan, Zhiming Yuan, Yanfeng Yao

Last updated by author(s): Sep 18, 2023

Reporting Summary

Nature Portfolio wishes to improve the reproducibility of the work that we publish. This form provides structure for consistency and transparency in reporting. For further information on Nature Portfolio policies, see our Editorial Policies and the Editorial Policy Checklist.

Statistics

For all statistical analyses, confirm that the following items are present in the figure legend, table legend, main text, or Methods section.

n/a	Confirmed	
☐	☒	The exact sample size (*n*) for each experimental group/condition, given as a discrete number and unit of measurement
☐	☒	A statement on whether measurements were taken from distinct samples or whether the same sample was measured repeatedly
☐	☒	The statistical test(s) used AND whether they are one- or two-sided<br>*Only common tests should be described solely by name; describe more complex techniques in the Methods section.*
☒	☐	A description of all covariates tested
☒	☐	A description of any assumptions or corrections, such as tests of normality and adjustment for multiple comparisons
☐	☒	A full description of the statistical parameters including central tendency (e.g. means) or other basic estimates (e.g. regression coefficient) AND variation (e.g. standard deviation) or associated estimates of uncertainty (e.g. confidence intervals)
☐	☒	For null hypothesis testing, the test statistic (e.g. *F*, *t*, *r*) with confidence intervals, effect sizes, degrees of freedom and *P* value noted<br>*Give P values as exact values whenever suitable.*
☒	☐	For Bayesian analysis, information on the choice of priors and Markov chain Monte Carlo settings
☒	☐	For hierarchical and complex designs, identification of the appropriate level for tests and full reporting of outcomes
☒	☐	Estimates of effect sizes (e.g. Cohen's *d*, Pearson's *r*), indicating how they were calculated

Our web collection on statistics for biologists contains articles on many of the points above.

Software and code

Policy information about availability of computer code

Data collection	ImmunoSpot Software (Cellular Technology) and Pannoramic MIDI system (3DHISTECH, Budapest, Hungary).
Data analysis	GraphPad Prism 8.0 software, Mega11, caseViewer, imageJ.

For manuscripts utilizing custom algorithms or software that are central to the research but not yet described in published literature, software must be made available to editors and reviewers. We strongly encourage code deposition in a community repository (e.g. GitHub). See the Nature Portfolio guidelines for submitting code & software for further information.

Data

Policy information about availability of data

All manuscripts must include a data availability statement. This statement should provide the following information, where applicable:

- Accession codes, unique identifiers, or web links for publicly available datasets
- A description of any restrictions on data availability
- For clinical datasets or third party data, please ensure that the statement adheres to our policy

The data that support the findings of this study are available from the corresponding author upon reasonable request.

Research involving human participants, their data, or biological material

Policy information about studies with human participants or human data. See also policy information about sex, gender (identity/presentation), and sexual orientation and race, ethnicity and racism.

Reporting on sex and gender	None
Reporting on race, ethnicity, or other socially relevant groupings	None
Population characteristics	None
Recruitment	None
Ethics oversight	None

Note that full information on the approval of the study protocol must also be provided in the manuscript.

Field-specific reporting

Please select the one below that is the best fit for your research. If you are not sure, read the appropriate sections before making your selection.

☒ Life sciences ☐ Behavioural & social sciences ☐ Ecological, evolutionary & environmental sciences

For a reference copy of the document with all sections, see nature.com/documents/nr-reporting-summary-flat.pdf

Life sciences study design

All studies must disclose on these points even when the disclosure is negative.

Sample size	The minimal replicate number for animal experiments was chosen to be n≥3. The control group size of n=3 in hamster challenge studies is quite small for nipah virus studies. We have shown clinical scores (Supplementary Fig. 3) over time for the corresponding challenge groups to strengthen our findings.
Data exclusions	Data were not excluded from analysis.
Replication	The minimal replicate number for animal experiments was chosen to be n≥3. All attempts of replication were successful and gave similar results.
Randomization	Female BALB/c mice and Syrian hamsters used in this study were selected randomly.
Blinding	Animal immunization and challenge experiments do not allow for blinding. The individuals performing HE and tissue sample analysis were unaware of the sample identity and obtained results consistent with the non-double-blind analysis.

Reporting for specific materials, systems and methods

We require information from authors about some types of materials, experimental systems and methods used in many studies. Here, indicate whether each material, system or method listed is relevant to your study. If you are not sure if a list item applies to your research, read the appropriate section before selecting a response.

Materials & experimental systems

n/a	Involved in the study	
☐	☒	Antibodies
☐	☒	Eukaryotic cell lines
☒	☐	Palaeontology and archaeology
☐	☒	Animals and other organisms
☒	☐	Clinical data
☒	☐	Dual use research of concern
☒	☐	Plants

Methods

n/a	Involved in the study	
☒	☐	ChIP-seq
☒	☐	Flow cytometry
☒	☐	MRI-based neuroimaging

Antibodies

Antibodies used	mouse antibody against NiV-G serum at 1:500 dilutions (prepared by our laboratory)

Antibodies used	mouse Anti-GAPDH antibody [6C5] - Loading Control (Abcam, ab8245,diluted 1:5000)<br>HRP-conjugated goat anti-mouse IgG (Abcam, ab6789,diluted 1:20000), HRP-conjugated goat anti-mousegA (Abcam, ab97235,diluted 1:10000)<br>HRP-conjugated goat anti- hamster IgG (Abcam, ab6892,diluted 1:15000)<br>HRP-conjugated secondary anti-mice IgG (Proteintech, SA00001-1, diluted 1:2000)<br>a rabbit anti-NiV N protein antibody at 1:3000 dilutions (prepared by our laboratory)<br>HRP-conjugated goat anti- rabbit IgG (SeraCare, Cat No.5220-0336) at 1:500 dilutions
Validation	mouse Anti-GAPDH antibody [6C5],HRP-conjugated goat anti-mouse IgG ,HRP-conjugated goat anti-mousegA,HRP-conjugated goat anti- hamster IgG,HRP-conjugated secondary anti-mice IgG and HRP-conjugated goat anti- rabbit IgG has been validated (see website),mouse antibody against NiV-G serum and rabbit anti-NiV N protein antibody has been validated by our laboratory

Eukaryotic cell lines

Policy information about cell lines and Sex and Gender in Research

Cell line source(s)	HEK293T (Human,ATCC: ACS-4500), HEK293 (Human,ATCC: CRL-1573) and Vero E6 (monkey, ATCC: CRL-1586) .
Authentication	None of the cell lines have been authenticated.
Mycoplasma contamination	Cell lines were tested for mycoplasma contamination.
Commonly misidentified lines (See ICLAC register)	No commonly misidentified cell lines were used.

Animals and other research organisms

Policy information about studies involving animals; ARRIVE guidelines recommended for reporting animal research, and Sex and Gender in Research

Laboratory animals	Female BALB/c mice (6-8 weeks old, female) and Syrian hamsters (5-6 weeks old, female) were purchased from Vital River Laboratories (Beijing, China).
Wild animals	No wild animals were used in this study.
Reporting on sex	We used all female mice for this study and did not set up different sex groups for comparative studies.
Field-collected samples	No field-collected samples were used in this study.
Ethics oversight	The animal experiments were approved by the Animal Ethics Committee of the Wuhan Institute of Virology, Chinese Academy of Sciences (approval number: WIVA21202104). All animal experiments involving NiV were executed in the animal biosafety level 4 (ABSL-4) facility at the National Biosafety Laboratory (Wuhan), Chinese Academy of Sciences.

Note that full information on the approval of the study protocol must also be provided in the manuscript.
