# Supplementary material for: Both chimpanzee adenovirus-vectored and DNA vaccines induced long-term immunity against Nipah virus infection
Source: NPJ Vaccines. 2023 Nov 4;8:170. doi: 10.1038/s41541-023-00762-3 (PMC10625554; doi:10.1038/s41541-023-00762-3)
Supplement: Supplementary file 2 — Supplementary information [file 41541_2023_762_MOESM2_ESM.pdf]

a **G sequence**

MPTESKKVRFENTASDKGKNPSKVIKSYYGTMDIKKINEGLLDSKILSAFNTVIALLG-
SIVIIVMNIMIIQNYTRSTDNQAMIKDALQSIQQQIKGLADKIGTEIGPKVSLIDTSSTITIP
ANIGLLGSKISQSTASINENVNEKCKFTLPPLKIHECNISCPNPLPFREYKPQTEGVSN
LVGLPNNICLQKTSNQILKPKLISYTLPVVGQSGTCITDPLLAMDEGYFAYSHLEKIGS
CSRGVSKQRIIGVGEVLDRGDEVPSLFMTNVWTPSNPNTVYHCSAVYNNEFYYVLCA
VSVVGDPILNSTYWSGSLMMTRLAVKPKNNGESYNQHQFALRNIEKGKYDKVMPYG
PSGIKQGDTLYFPAVGFLVRTEFKYNDSNCPIAKCQYSKPENCRLSMGIRPNSHYILR
SGLLKYNLSDEENSKIVFIEISDQRLSIGSPSKIYDSLGQPVFYQASFSWDTMIKFGDV
QTVNPLVVNWRNNTVISRPGQSQCPRFNTCPEVCWEGVYNDAFLIDRINWISAGVFL
DSNQTAENPVFTVFKDNEVLYRAQLASEDTNAQKTITNCFLLKNKIWCISLVEIYDTG
DNVIRPKLFAVKIPEQCT

b **Phylogenetic tree**

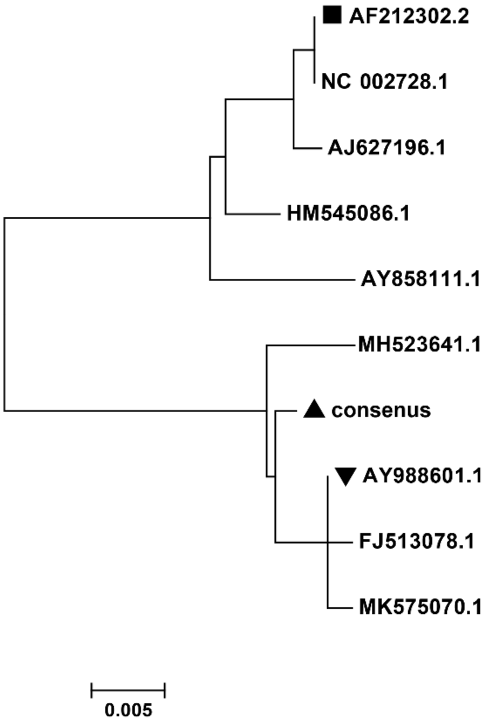

Supplementary Fig. 1 Full-length NiV G amino acid sequence and the phylogenetic tree.

a. The consensus G protein sequence in the vaccine study. **b.** The phylogenetic tree results of the G amino acid sequence in this study (black triangle) was compared to

other G amino acid sequences in GenBank. Black square (AF212302.2) and black inverted triangle (AY988601.1) were used for the virus challenge experiment.

NiV-Bangladesh

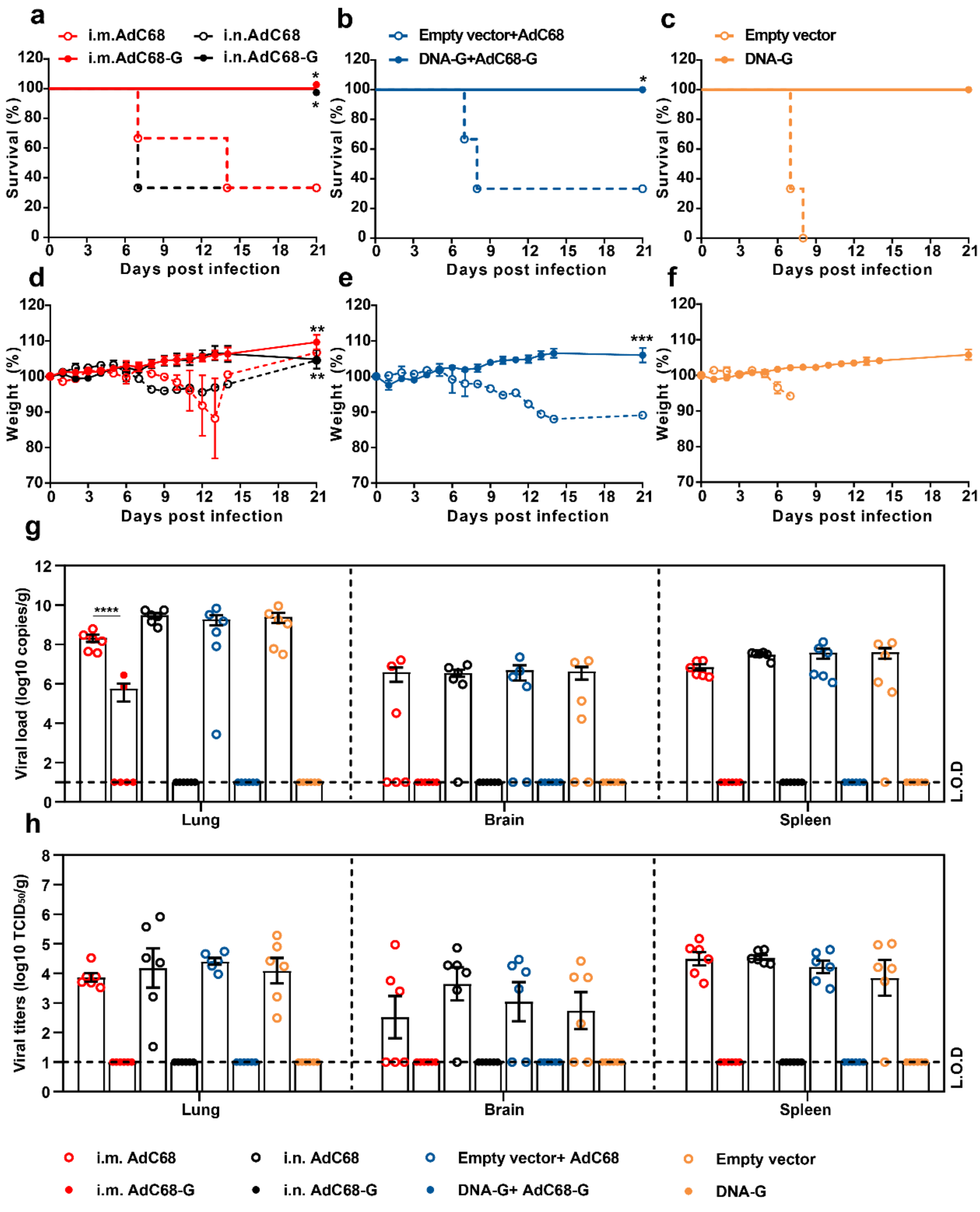

9

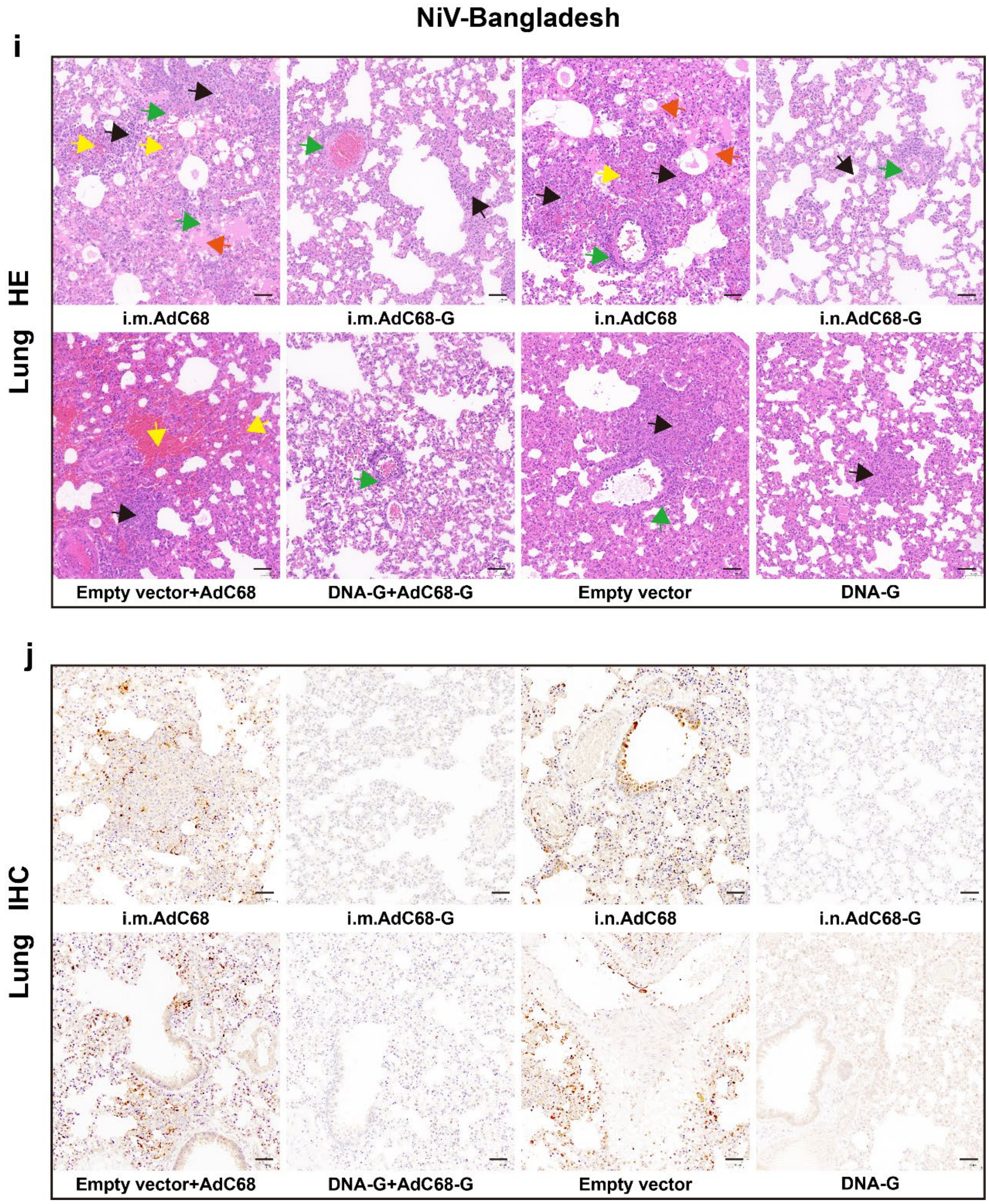
NiV-Bangladesh
i
Lung HE
i.m.AdC68
i.m.AdC68-G
i.n.AdC68
i.n.AdC68-G
Empty vector+AdC68
DNA-G+AdC68-G
Empty vector
DNA-G
j
Lung IHC
i.m.AdC68
i.m.AdC68-G
i.n.AdC68
i.n.AdC68-G
Empty vector+AdC68
DNA-G+AdC68-G
Empty vector
DNA-G

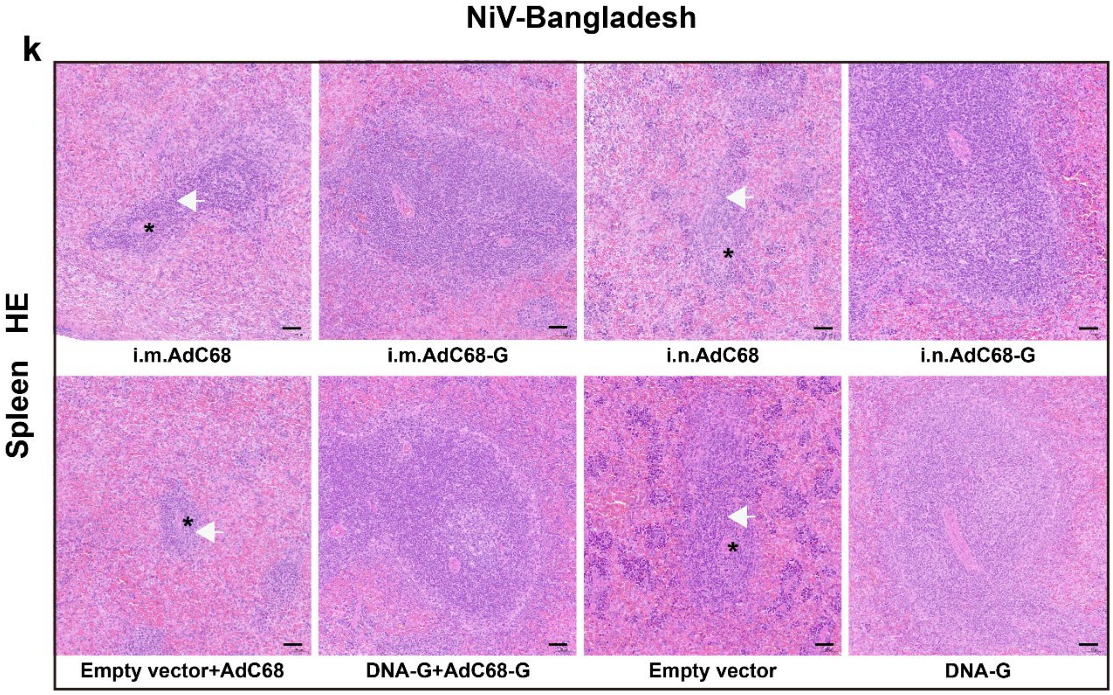

Supplementary Fig. 2 AdC68-G and DNA vaccines protected Syrian golden hamsters from lethal NiV Bangladesh challenges.

a-f. Survival **(a-c)** and weight change **(d-f)** of Syrian hamsters challenged with the NiV Bangladesh strain. **g-h.** Viral loads in the hamster lungs, brain, and spleen at 5 d.p.i. quantified by quantitative PCR with reverse transcription (RT–qPCR) **(g)** and live virus titration **(h)**. **i.** Lung tissue sections were stained with hematoxylin and eosin (HE). Marked bronchointerstitial pneumonia with inflammatory cell infiltration or necrosis (black arrows), hemorrhage (yellow arrows), vasculitis (green arrows), fibrous exudation (blue arrows), and edema (orange arrows) in the lung tissue of control animals. Mild individual symptoms were observed in vaccinated animals. **j.** Lung tissue sections were stained with an antibody against the NiV N antigen, which was visible as red–brown staining (IHC). No immunoreactivity was found in vaccinated animals, whereas multifocal immunoreactivity could be found in the lung tissue of control

animals. **k.** Spleen tissue sections were stained with hematoxylin and eosin (HE). Loss of normal splenic architecture, with decreased lymphocyte numbers in white pulp (white arrows); the asterisk indicates the central artery. Data are presented with the group mean± SEM. Two-tailed unpaired Student's t-tests were conducted to compare differences between two experimental groups. $^*p < 0.05$, $^{***}p < 0.001$, $^{****}p < 0.0001$. Error bars indicate 95% confidence intervals. L.O.D. represents the limit of detection. Bar scale, 50 μm.

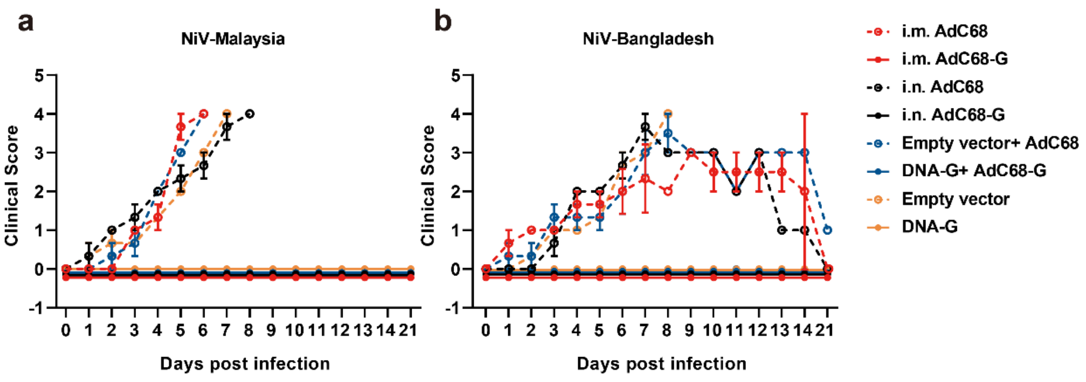

Illness scoring
0- no symptoms;
1- loss of body condition;
2- ruffled fur, slowed activity, loss of body condition, moderately labored breathing, and hunched posture;
3- ruffled fur, obviously labored breathing and bleeding;
4-moribund and death.

Supplementary Fig. 3 AdC68-G and DNA vaccines reduce course of disease in Syrian golden hamsters challenged with lethal NiV Malaysia and Bangladesh.

a. The clinical score of the hamster infected with Malaysia strain. **b.** The clinical score of the hamster infected with Bangladesh strain. The severity of the animals' illnesses was scored daily, as follows: 0 - no symptoms; 1 - loss of body condition; 2 - ruffled fur, slowed activity, loss of body condition, moderately labored breathing and hunched posture; 3 - ruffled fur, obviously labored breathing and bleeding; 4 - moribund and death. Data are presented with the group mean ± SEM. Error bars indicate 95% confidence intervals.

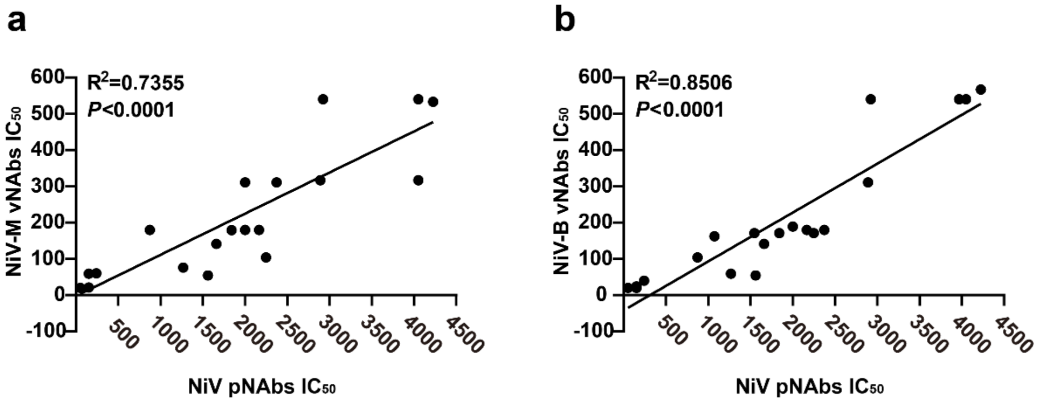

Supplementary Fig. 4 Comparison of the live Nipah virus neutralization and pseudovirus neutralization assays in mice.

a-b. Scatter plots of the live NiV-M **(a)** or NiV-B **(b)** neutralization assay-derived neutralization antibosys IC_{50} and NiV pseudovirus neutralization assay-derived neutralization antibosys IC_{50} values.

a

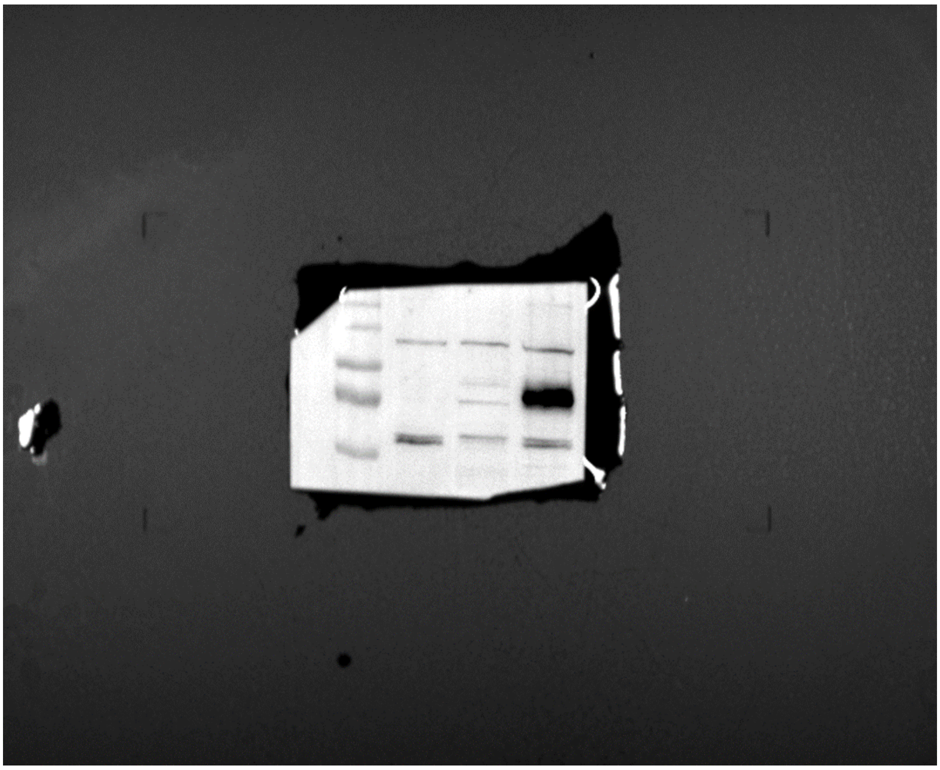

b

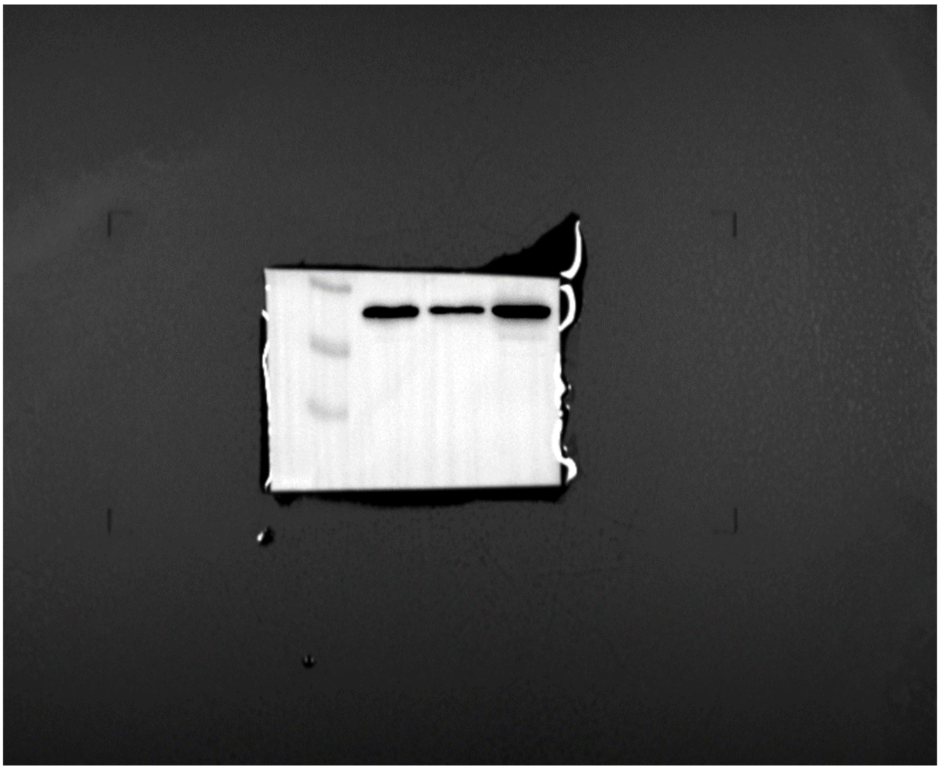

c

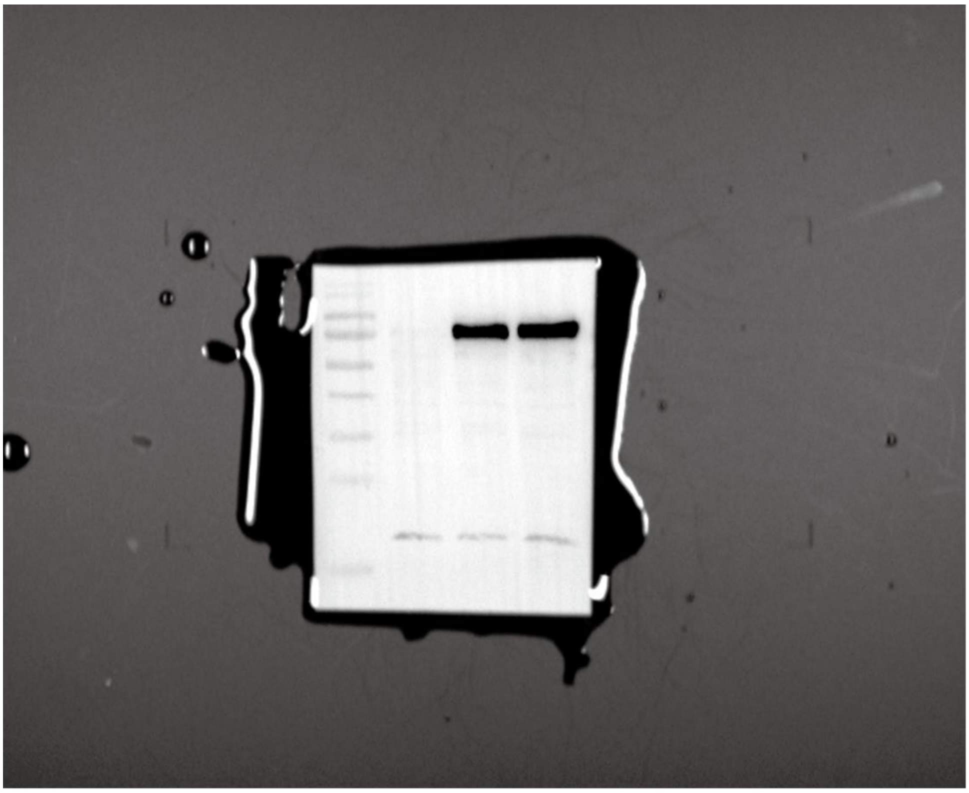

d

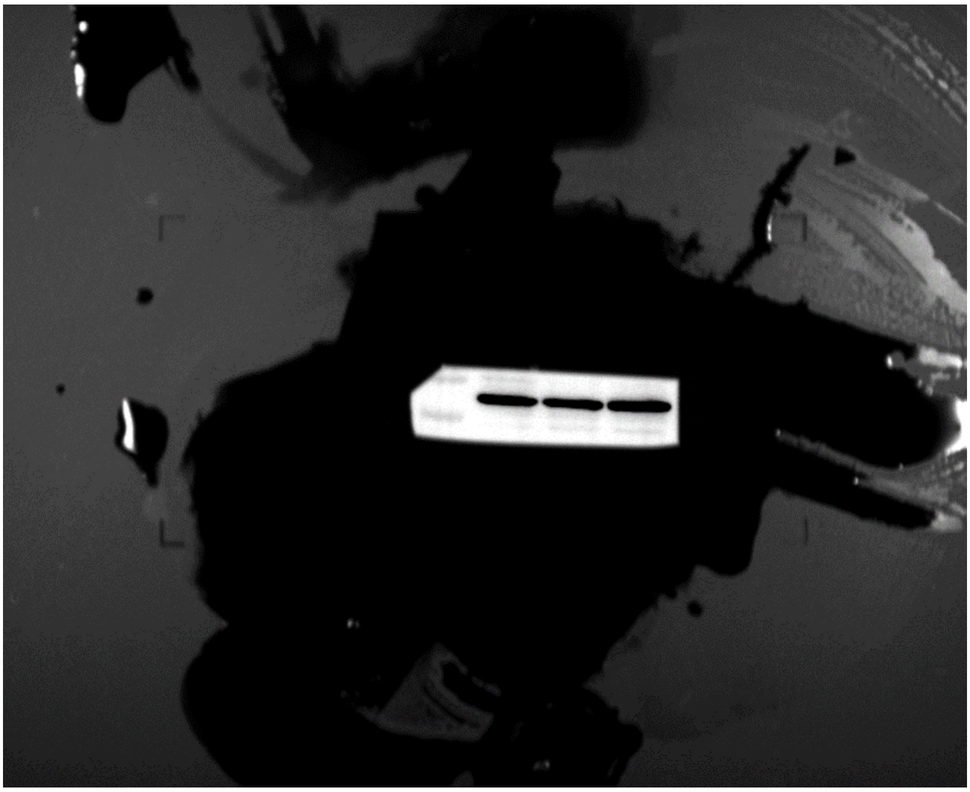

Supplementary Fig. 5 Uncropped and unprocessed scans corresponds to the Figs. 1d, e

a. Fig.1d NiV-G uncropped and unprocessed scans. **b.** Fig.1d GAPDH uncropped and unprocessed scans. **c.** Fig. 1e NiV-G uncropped and unprocessed scans (The first three bands). **d.** Fig. 1e GAPDH uncropped and unprocessed scans (The first three bands).
